# An orally available compound suppresses glucagon hypersecretion and normalizes hyperglycemia in type 1 diabetes

**DOI:** 10.1172/jci.insight.172626

**Published:** 2024-01-23

**Authors:** Farzad Asadi, Subhadra C. Gunawardana, Roland E. Dolle, David W. Piston

**Affiliations:** 1 Department of Cell Biology and Physiology and; 2 Center for Drug Discovery, Washington University School of Medicine, St. Louis, Missouri, USA.

**Keywords:** Endocrinology, Diabetes

## Abstract

Suppression of glucagon hypersecretion can normalize hyperglycemia during type 1 diabetes (T1D). Activating erythropoietin-producing human hepatocellular receptor type-A4 (EphA4) on α cells reduced glucagon hypersecretion from dispersed α cells and T1D islets from both human donor and mouse models. We synthesized a high-affinity small molecule agonist for the EphA4 receptor, WCDD301, which showed robust plasma and liver microsome metabolic stability in both mouse and human preparations. In islets and dispersed islet cells from nondiabetic and T1D human donors, WCDD301 reduced glucagon secretion comparable to the natural EphA4 ligand, Ephrin-A5. In diabetic NOD and streptozotocin-treated mice, once-daily oral administration of WCDD301 formulated with a time-release excipient reduced plasma glucagon and normalized blood glucose for more than 3 months. These results suggest that targeting the α cell EphA4 receptor by sustained release of WCDD301 is a promising pharmacologic pathway for normalizing hyperglycemia in patients with T1D.

## Abstract 2

<p>WCDD301 is an oral drug that agonizes EphA4 receptors in &alpha;-cells to suppress glucagon hypersecretion and normalize hyperglycemia in type 1 diabetes.</p>

## Graphical abstract

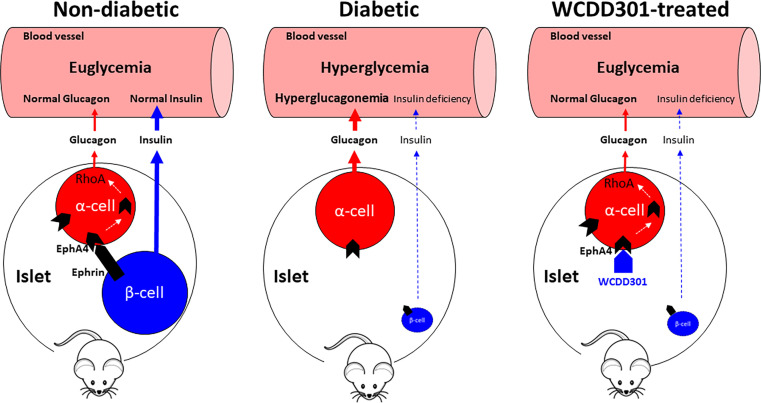

## Introduction

Type 1 diabetes (T1D), characterized by severe insulin deficiency, is widespread in both pediatric and adult populations (1). Individuals with T1D require injection of insulin, an unstable peptide, to treat hyperglycemia and prevent death. The drawbacks of insulin treatment include invasive administration along with risks of hypoglycemia (2) and weight gain (3). Alternatives to insulin-only therapy could improve the quality of life for people with T1D. Destruction of pancreatic β cells in T1D not only brings about glucose intolerance but also disrupts the normal glucagon response of α cells to hypoglycemia (4). The loss of β cells leads to abnormal behavior of α cells, causing glucagon hypersecretion that exacerbates hyperglycemia through stimulation of gluconeogenesis and glycogenolysis (5, 6). Accordingly, a glucagon-centric hypothesis proposes that combating hyperglucagonemia can result in normalization of hyperglycemia in T1D (7).

Targeting hyperglucagonemia as a complementary therapeutic tactic for insulin treatment in T1D has resulted in several strategies, including brown adipose tissue transplantation (by an unknown mechanism, potentially through secreted factors’ effects on insulin receptor) (8–13) and use of glucagon receptor antagonists (14–17), antibodies (18), or chemical compounds (19). Among these approaches, treatment with glucagon receptor–competitive or –allosteric antagonists (16) in clinical trials has been marked by side effects, including α cell hyperplasia that exacerbates hyperglucagonemia and yields recurring of hyperglycemia (20), hyperlipidemia (21), elevation of plasma transaminases (22), and weight gain (23). Based on these observations, researchers explored suppression of glucagon secretion as a possible strategy for treating hyperglycemia in T1D (24–28). However, this strategy has been hindered by poor understanding of the mechanisms underlying the regulation of glucagon secretion, which remain a puzzle even 100 years after its discovery (27, 29–31).

Flow-sorting α cells or dispersing islets into single cells mimics the α cell T1D phenotype, where glucagon secretion increases as a function of glucose (32, 33). Reaggregation of α and β cells into pseudo-islets results in suppression of glucagon secretion (28), suggesting that physical contact between α and β cells may play a role in regulating glucagon secretion. EphrinA-EphA signaling is one mechanism that has been shown to mediate cell-cell contacts in the islet (32, 34, 35), with erythropoietin-producing human hepatocellular receptor type-A4 (EphA4) playing a central role in the α cell. The misregulation of glucagon secretion from dispersed α cells is accompanied by reduced F-actin intensity. Treating dispersed α cells with soluble Ephrin-A5 (a natural ligand of EphA4 receptors) restored normal F-actin intensity and glucagon secretion profiles (35). These findings suggest the presence of an axis between Ephrin-A5-EphA4 complex and F-actin in α cells toward regulation of glucagon secretion. In T1D when there is a lack of β cells in islets, this axis is compromised, which results in glucagon hypersecretion. Here, we describe the synthesis and use of an orally available EphA4 agonist, named WCDD301, that suppresses glucagon hypersecretion and restores euglycemia in T1D mouse models.

## Results

Doxazosin has been reported as a potential agonist of EphA4 receptors (36), so we tested its efficacy in suppressing glucagon secretion from human and mouse α cells. However, this compound increased glucagon secretion over a range of concentrations (Supplemental Figure 1, A and B; supplemental material available online with this article; https://doi.org/10.1172/jci.insight.172626DS1), and conjugate derivatives of doxazosin with additional hydrophilic (lysine or tyrosine) or hydrophobic (proline) amino acids also increased glucagon secretion (Supplemental Figure 1, C–E). By considering the structural properties of doxazosin and another reported EphA4 agonist, 123C4 (37), we designed a series of small noncyclic molecules and tested their effects on glucagon secretion. The results of these experiments led to the identification of a promising agonist that we named WCDD301.

### WCDD301 is a small molecule that competitively binds to the EphA4 receptor.

**Figure 1:**
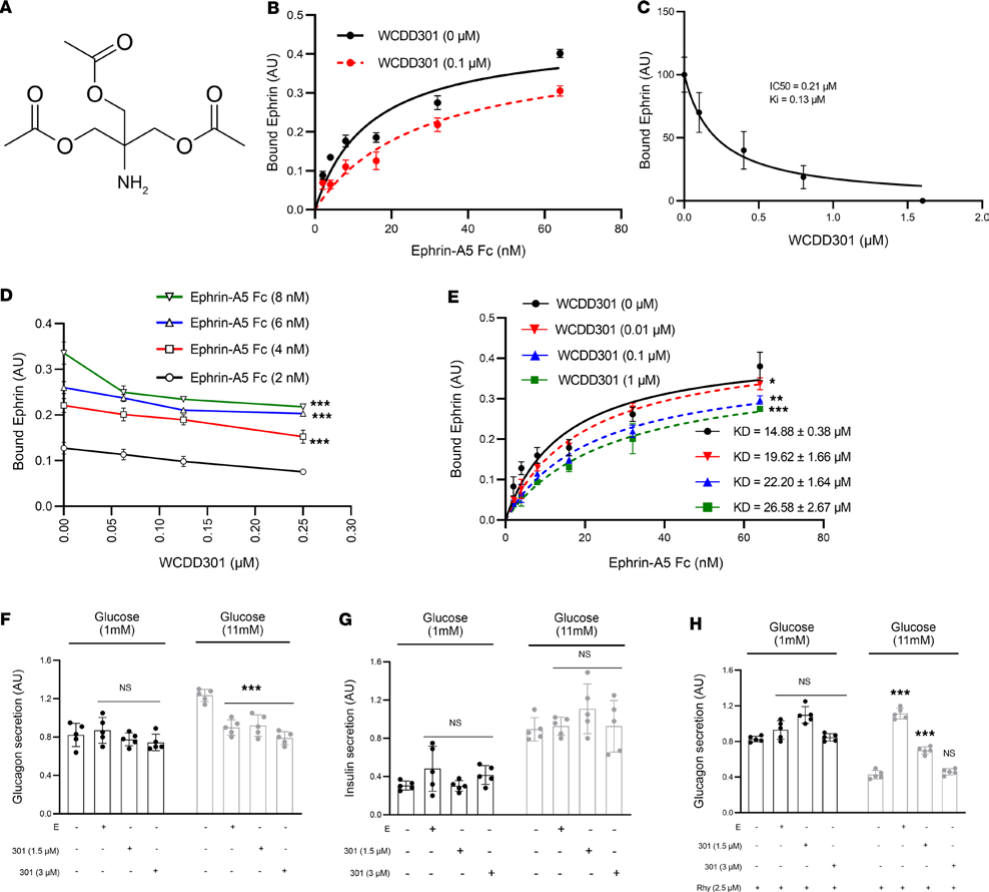
High binding affinity of WCDD301 for EphA4 receptor in a competitive way that suppresses glucagon secretion without side effect on insulin secretion. (**A**) Structural formula of WCDD301. (**B**) Determining binding affinity of WCDD301 for EphA4 without global fitting; each curve demonstrates best fit values of 3 individual replicates. (**C**) Determining binding affinity of WCDD301 for EphA4 via Cheng-Prusoff equation; each curve demonstrates best fit values of 3 individual replicates. (**D**) Demonstrating competition between WCDD301 and Ephrin-A5 Fc concentrations for binding to a fixed level of EphA4 (3 μg/mL) in ELISA system via AUC analysis. Values (mean ± SD) of AUC at 2 nM (0.025 ± 0.0011 AU), 4 nM (0.0469 ± 0.0014 AU), 6 nM (0.0554 ± 0.0006 AU), and 8 nM (0.0617 ± 0.0011 AU) of WCDD301 were compared; *** indicates difference of *P* < 0.001 between each successive concentration of Ephrin-A5 Fc (*n* = 3). (**E**) Competition between WCDD301 and Ephrin-A5 Fc for binding to a fixed level of EphA4 (3 μg/mL) through Michaelis-Menten kinetics; *K_i_* values (*n* = 3) of WCDD301 challenges were compared with the 0 μM of WCDD301. Relative secretion of (**F**) glucagon and (**G**) insulin in murine dispersed islet cells in the presence and absence of WCDD301 or Ephrin-A5 Fc (**E**). Values of treatment groups were compared with the respective control; each dot demonstrates values (mean ± SD) of a single mouse (*n* = 5). (**H**) Secretion of glucagon in murine dispersed islet cells following cotreatment of WCDD301 with an EphA4 antagonist, rhyncophylline (Rhy). Values of treatment groups were compared with the corresponding controls; each dot demonstrates values (mean ± SD) of a single mouse (*n* = 5). In all experiments, values (mean ± SEM) were compared using 1-way ANOVA, α = 0.05. *P < 0.05, **P < 0.01, ***P < 0.001.

WCDD301 is a symmetric achiral amine triacetate with MW 247.11 Da (Figure 1A). WCDD301 displays a high binding affinity for EphA4 receptor with a *K_i_* ≤ 0.13 μM (Figure 1, B and C). This compound functions as an EphA4 agonist and competes with the natural ligand, Ephrin-A5, as demonstrated by area under the curve (AUC) analysis (Figure 1D). The profile of competition between WCDD301 and Ephrin-A5 for binding to the EphA4 receptor follows Michaelis-Menten kinetics (Figure 1E).

### WCDD301 suppresses glucagon secretion in murine α cells by binding to EphA4 receptor.

In murine dispersed islet cells, α cells responded to the switch from 1 mM to 11 mM glucose by increasing glucagon secretion by about 50%, consistent with previous studies (33, 38, 39). Treatment with WCDD301 suppressed glucagon secretion at 11 mM glucose (Figure 1F) without significant effects on insulin secretion (Figure 1G). Arginine treatment significantly increased glucagon secretion, but its effect was suppressed by the application of either soluble Ephrin-A5 or WCDD301 at both 1 mM and 11 mM glucose (Supplemental Figure 2). Inhibition of the EphA4 receptor using a competitive inhibitor (rhyncophylline) abolished the suppressive effect of both Ephrin-A5 and 1.5 μM WCDD301 on glucagon secretion. However, by increasing the WCDD301 concentration to 3 μM, it outcompeted the inhibitor (Figure 1H) but still showed no significant effect on insulin secretion (Supplemental Figure 3).

### WCDD301 suppresses glucagon secretion by α cells from both healthy and type 1 diabetic human donors.

**Figure 2:**
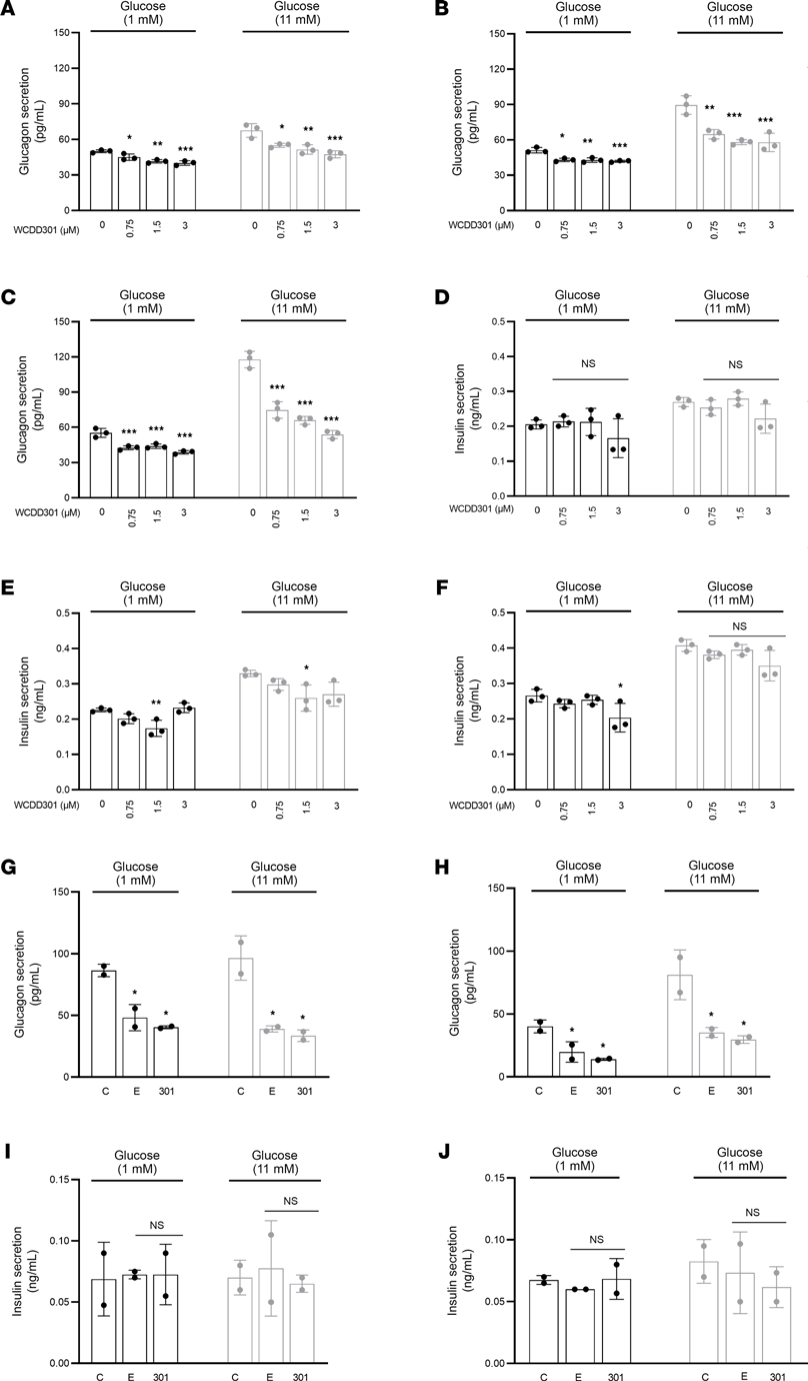
WCDD301 suppresses glucagon secretion from human α cells in both healthy and type 1 diabetic participants. Glucagon secretion from healthy human donor (*n* = 3) dispersed islet cells following (**A**) 1 hour, (**B**) 3 hours, or (**C**) 6 hours of exposure to WCDD301. Insulin secretion from healthy human dispersed islet cells following (**D**) 1 hour, (**E**) 3 hours, or (**F**) 6 hours of exposure to WCDD301. Each dot demonstrates values (mean ± SD) of an individual. In each panel, values (mean ± SEM) were compared with the respective controls using 1-way ANOVA; *P < 0.05, **P < 0.01, ***P < 0.001. Secretion of glucagon in vehicle-treated (C, control) cells and cells treated with Ephrin-A5 Fc (**E**) or WCDD301 in (**G**) isolated islets and (**H**) dispersed islet cells of 2 donors with T1D. Secretion of insulin in (**I**) isolated islets and (**J**) dispersed islet cells of the donors with T1D. Each dot demonstrates mean values of the selected islets or dispersed islet cells for each subject. In each panel, values (mean ± SEM) were compared with the respective controls based on the level of changes. *remarkable changes of *P* < 0.05.

Dispersed human donor islet cells showed dose-dependent and time-dependent suppression of glucagon secretion following 1 hour (Figure 2A), 3 hours (Figure 2B), or 6 hours (Figure 2C) of exposure to WCDD301 in both 1 mM and 11 mM glucose culture. In these experiments, WCDD301 showed a transient effect on insulin secretion (Figure 2, D–F) but no effect on somatostatin secretion (Supplemental Figure 4, A–C). Profiling of glucagon secretion from islets or dispersed islet cells (29) showed that WCDD301 reduced glucagon secretion from islets (Figure 2G) and dispersed islet cells (Figure 2H) from donors with long-standing T1D with no effect on the minimal residual insulin secretion from islets (Figure 2I) or dispersed islet cells (Figure 2J).

### WCDD301 enhances intracellular intensities of EphA4 and F-actin.

**Figure 3:**
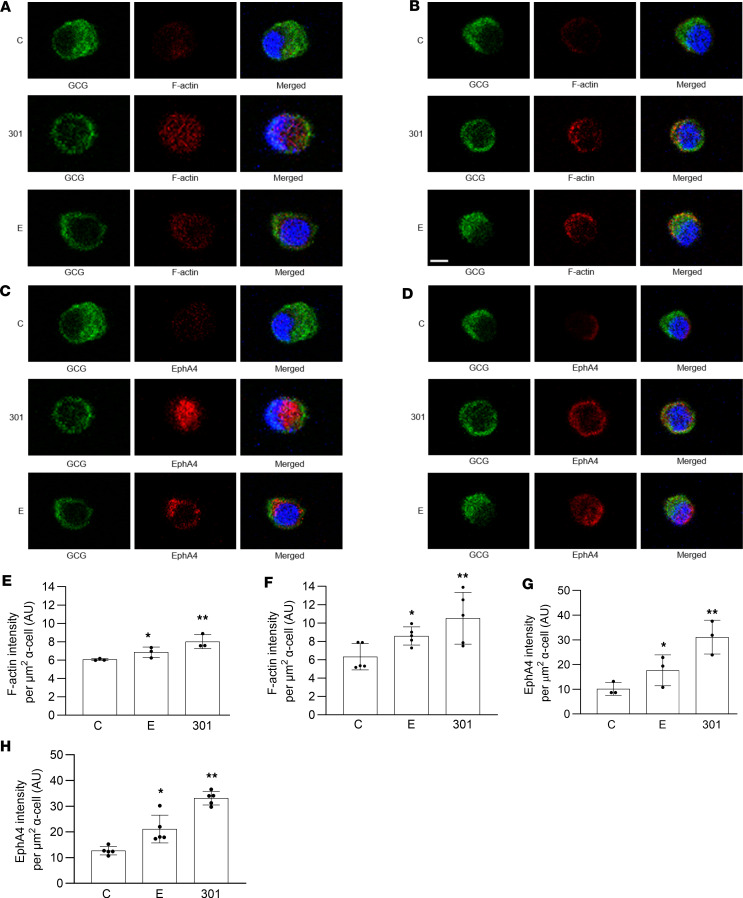
WCDD301 enhances intracellular F-actin and EphA4 intensities in both human and mouse dispersed islet cells. (**A**) Immunostained images of human dispersed islet cells for F-actin and glucagon in vehicle-treated (C, control) and cells treated with WCDD301 or Ephrin-A5 Fc (E). (**B**) Immunostained images of mouse dispersed islet cells for F-actin and glucagon in C, 301, and E groups. (**C**) Immunostained images of human dispersed islet cells for EphA4 and glucagon in C, 301, and E groups. (**D**) Immunostained images of mouse dispersed islet cells for EphA4 and glucagon in C, 301, and E groups. (**E**) Comparison of F-actin intensity among C, 301, and E groups of human cells. (**F**) Comparison of F-actin intensity among C, 301, and E groups of mouse cells. (**G**) Comparison of EphA4 intensity among C, 301, and E groups of human cells. (**H**) Comparison of EphA4 intensity among C, 301, and E groups of mouse cells. On the scatter bar plot, each dot represents values (mean ± SD) of 1 mouse or human. Values (mean ± SEM) of treatment groups (*n* = 3 human, and *n* = 5 mouse) were compared with the respective controls using 1-way ANOVA, α = 0.05. *P < 0.05, **P < 0.01. Scale bar: 10 μm.

**Figure 4:**
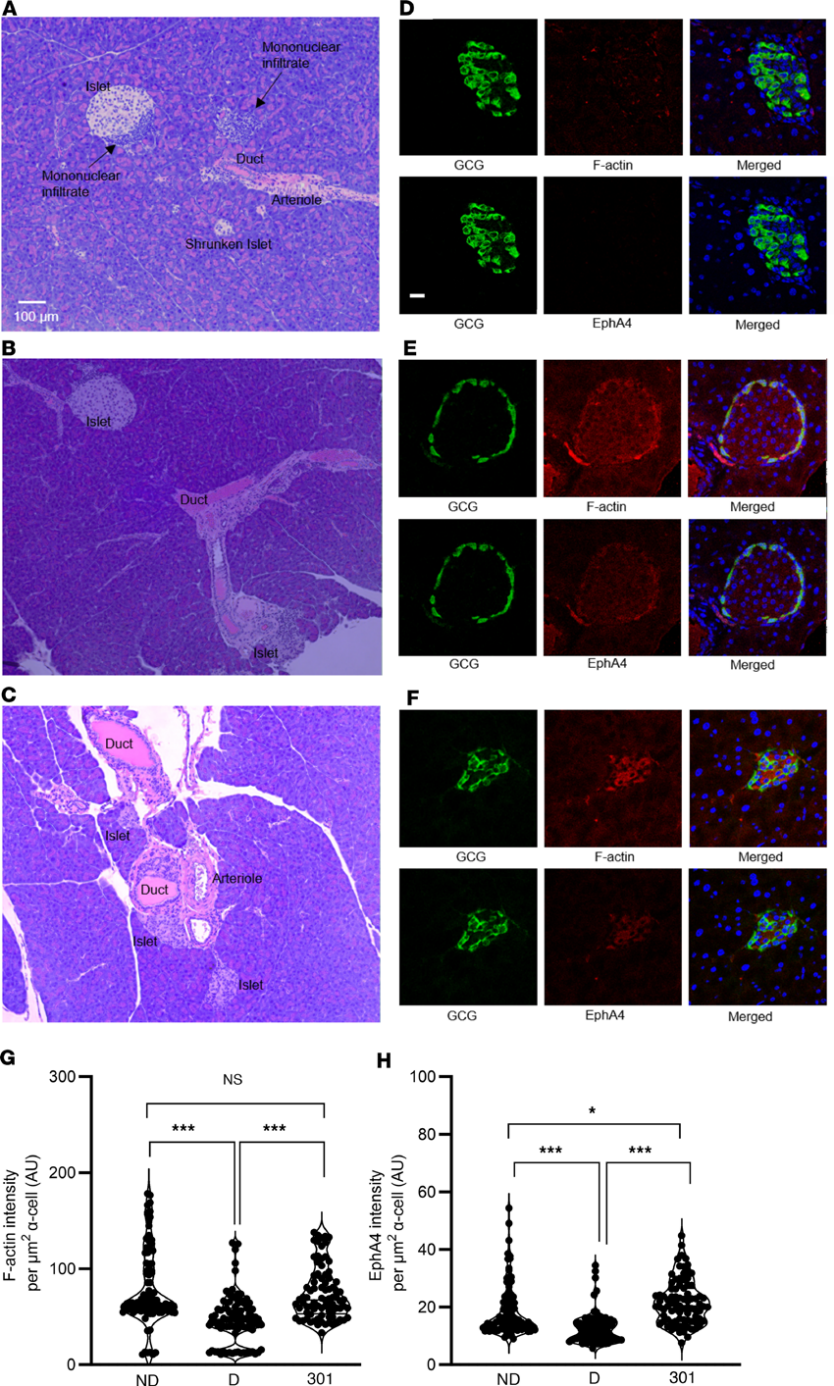
WCDD301 recovers mononuclear cell infiltrate and restores F-actin and EphA4 intensities in islets of NOD diabetic mice. Hematoxylin-Eosin staining of pancreatic islets in (**A**) diabetic, (**B**) nondiabetic, and (**C**) WCDD301-treated mice. Immunostaining of islets for glucagon, F-actin, and EphA4 in (**D**) diabetic, (**E**) nondiabetic, and (**F**) WCDD301-treated mice (scale bar: 20 μm). Quantitation of (**G**) F-actin and (**H**) EphA4 signals in α cells of nondiabetic, diabetic, and WCDD301-treated mice. Values (mean ± SEM; *n* = 3) compared among groups using 1-way ANOVA. *P < 0.05, ***P < 0.001.

WCDD301 treatment increased intracellular F-actin density in α cells as assayed by immunostaining of dispersed islet cells from human donors (Figure 3, A and E), similar to that observed following Ephrin-A5 treatment (35). These cells also showed increased intracellular EphA4 intensity in treatment with WCDD301 (Figure 3, C and G). Immunostaining of dispersed mouse islet cells likewise showed increased intracellular F-actin intensity in treated cells with WCDD301 (Figure 3, B and F). The increased F-actin signal correlated with increased EphA4 intensity (Figure 3, D and H) and reduced shedding of EphA4 into the cell culture medium (Supplemental Figure 4D). Hematoxylin-Eosin staining of islets from diabetic NOD mice (Figure 4A) showed a high level of mononuclear cell infiltration in and around islets and edema in exocrine pancreas compared with nondiabetic mice (Figure 4B). However, there was no mononuclear infiltration or apparent edema in the WCDD301-treated mice (Figure 4C). Immunofluorescence images showed dimmed F-actin and EphA4 signals in diabetic NOD mouse islets (Figure 4D) compared with either nondiabetic (Figure 4E) or WCDD301-treated mice (Figure 4F). Quantification revealed significant suppression (*P* < 0.001) in F-actin (Figure 4G) and EphA4 (Figure 4H) signals within α cells of diabetic mice (47.14 ± 4.70 AU; 12.64 ± 1.12 AU) compared with nondiabetic mice (79.14 ± 4.63 AU; 18.62 ± 1.09 AU). Normal levels of F-actin and EphA4 were recovered in WCDD301-treated mice (74.50 ± 4.80 AU; 21.84 ± 1.14 AU).

### WCDD301 is stable in mouse and human plasma and liver microsomes, and slow-release administration increases its plasma duration in mice.

**Figure 5:**
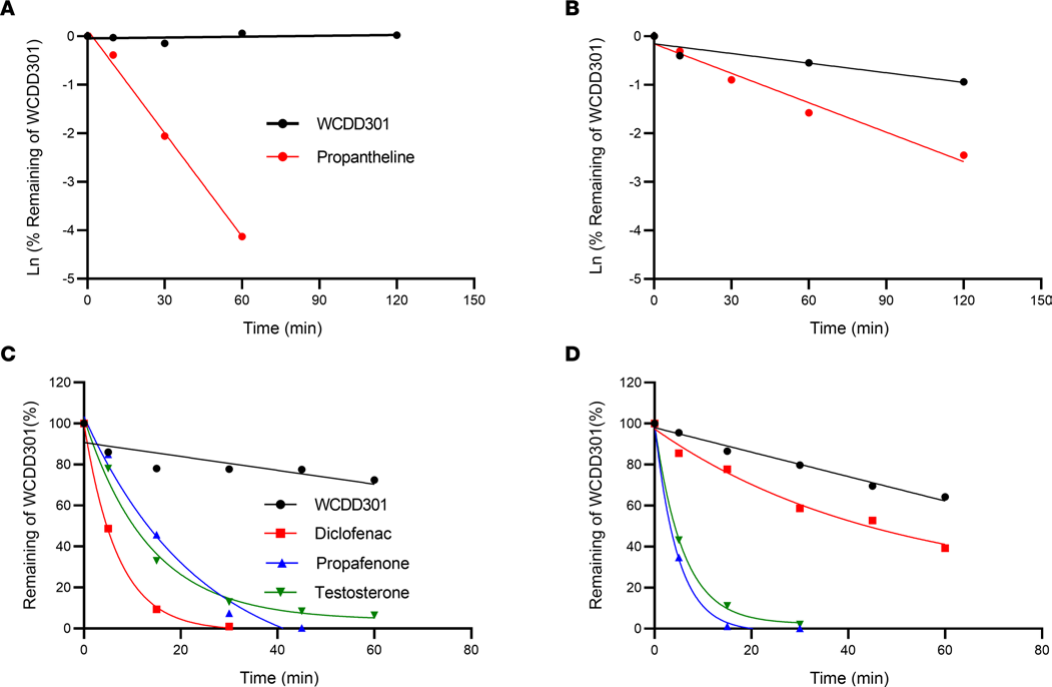
WCDD301 shows excellent in vitro metabolic stability. Stability of WCDD301 in pooled plasma of (**A**) human and (**B**) mouse compared with the positive control of propantheline. Stability of WCDD301 in hepatic microsomes of (**C**) human and (**D**) mouse compared with the positive controls of diclofenac, propafenone, and testosterone.

WCDD301 showed strong plasma stability compared with the control compound, propantheline, in both human (Figure 5A) and mouse (Figure 5B) plasma. As shown in Supplemental Table 1, WCDD301 stability in human plasma was > 2.7-fold higher than in mouse plasma. WCDD301 also demonstrated excellent microsomal stability (Figure 5C) compared with the controls of diclofenac, propafenone, and testosterone in an in vitro human liver microsome preparation. We observed similar stability in mouse liver microsomes (Figure 5D) compared with the 3 control compounds. The microsomal stability of WCDD301 (Supplemental Table 1) was > 1.5-fold higher in the human microsome preparation versus mouse. Pharmacokinetics in CD-1 mice revealed increased plasma concentrations of WCDD301 at 15 minutes following subcutaneous injection (Supplemental Figure 5A) compared with intravenous administration (Supplemental Figure 5B). This difference suggests that clearing of WCDD301 (putatively by excretion through the urine, as described below) allows the rate of its release into the plasma to determine its pharmacokinetics.

### WCDD301 normalizes blood glucose in mouse models.

**Figure 6:**
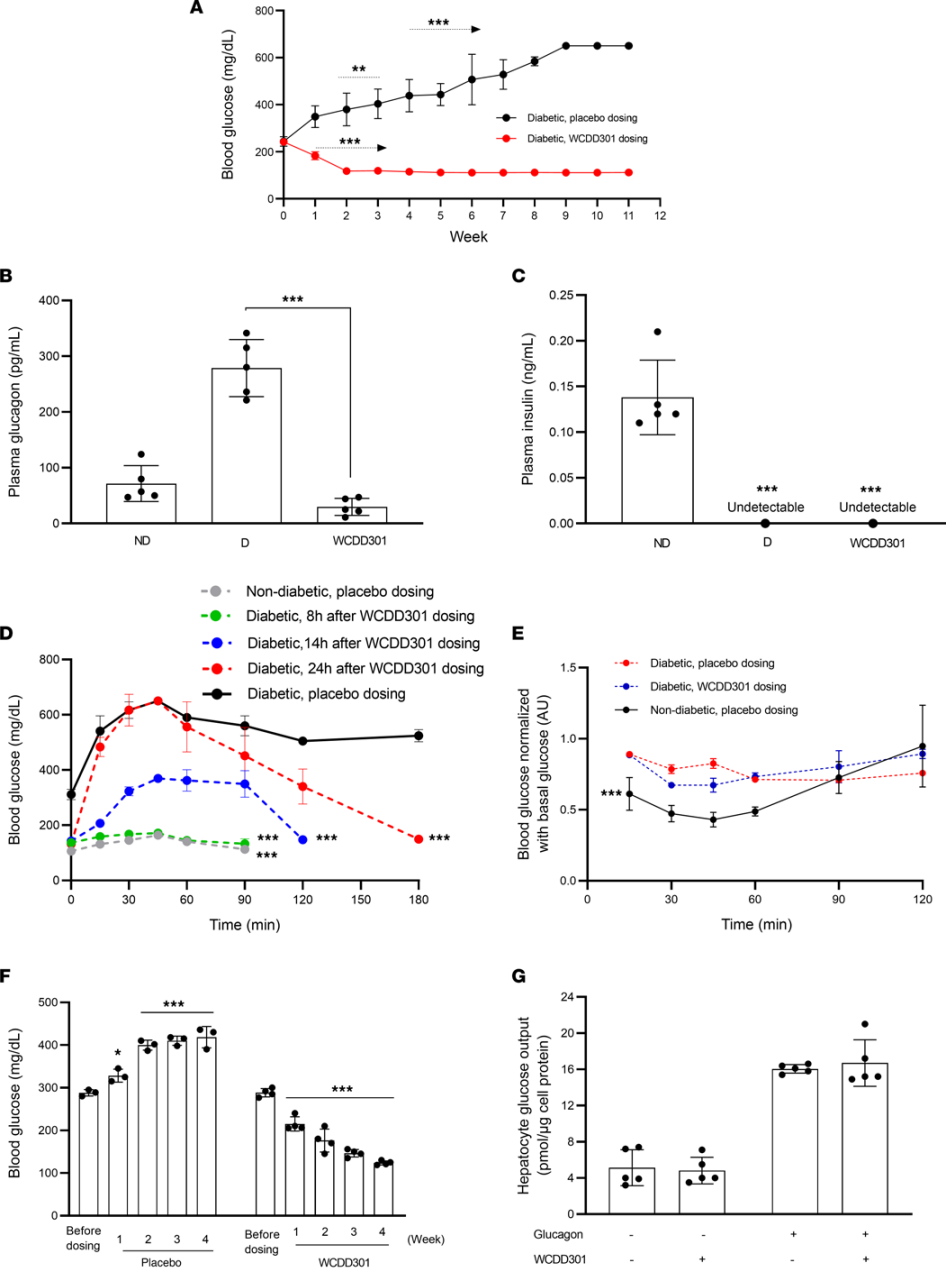
WCDD301 normalizes blood glucose levels in diabetic mouse models that is accompanied by improvement in glucose tolerance. (**A**) Treatment of diabetic NOD mice with moderate hyperglycemia using WCDD301 (*n* = 6–9, oral, 7.5 mg/kg) or placebo (*n* = 4) and monitoring blood glucose over 11 weeks. Values (mean ± SEM) in each week were compared with the values of before treatment (week 0) using 1-way ANOVA. **P < 0.01, ***P < 0.001. Plasma (**B**) glucagon and (**C**) insulin following treatment of the NOD mice; 1-way ANOVA; ***P < 0.001, *n* = 5; ND, nondiabetic; D, diabetic. (**D**) Intraperitoneal glucose tolerance in WCDD301-treated NOD mice; values expressed as mean ± SD (*n* = 3) and areas under the curves compared among groups using 1-way ANOVA. ***P < 0.001 compared with the nondiabetic placebo-treated mice. (**E**) Insulin tolerance in WCDD301-treated NOD mice; values expressed as mean ± SD (*n* = 4) and areas under curves compared among groups using 1-way ANOVA; ***P < 0.001 compared with the diabetic placebo-treated or diabetic WCDD301-treated mice. (**F**) Treatment of STZ-induced diabetic mice with early severe hyperglycemia using WCDD301 (10 mg/kg) and monitoring blood glucose over 4 weeks. Values of the placebo treated (*n* = 3) or WCDD301 treated (*n* = 4) in each week were compared with the corresponding values of before dosing using 1-way ANOVA; ***P < 0.001. (**G**) Glucose output from primary mouse hepatocytes in the presence or absence of 100 nM glucagon and/or 3 μM WCDD301 (*n* = 5). Each dot represents mean values of 1 mouse. Values of WCDD301-treated groups compared with the corresponding control using *t* test, α = 0.05.

When blood glucose levels of the diabetic NOD mice reached moderate hyperglycemia (200–250 mg/dL), once-daily oral dosing of the formulated WCDD301 in SAIB-Agar significantly reduced blood glucose levels within a week of administration. Continuing this daily treatment normalized blood glucose level during an 11-week follow-up compared with increasing hyperglycemia in the placebo-treated cohort (Figure 6A). To verify that these glycemic effects were due to WCDD301, we discontinued its administration for 48 hours, which resulted in hyperglycemia. Euglycemia returned after resuming the WCDD301 dosing (Supplemental Figure 6A).

**Figure 7:**
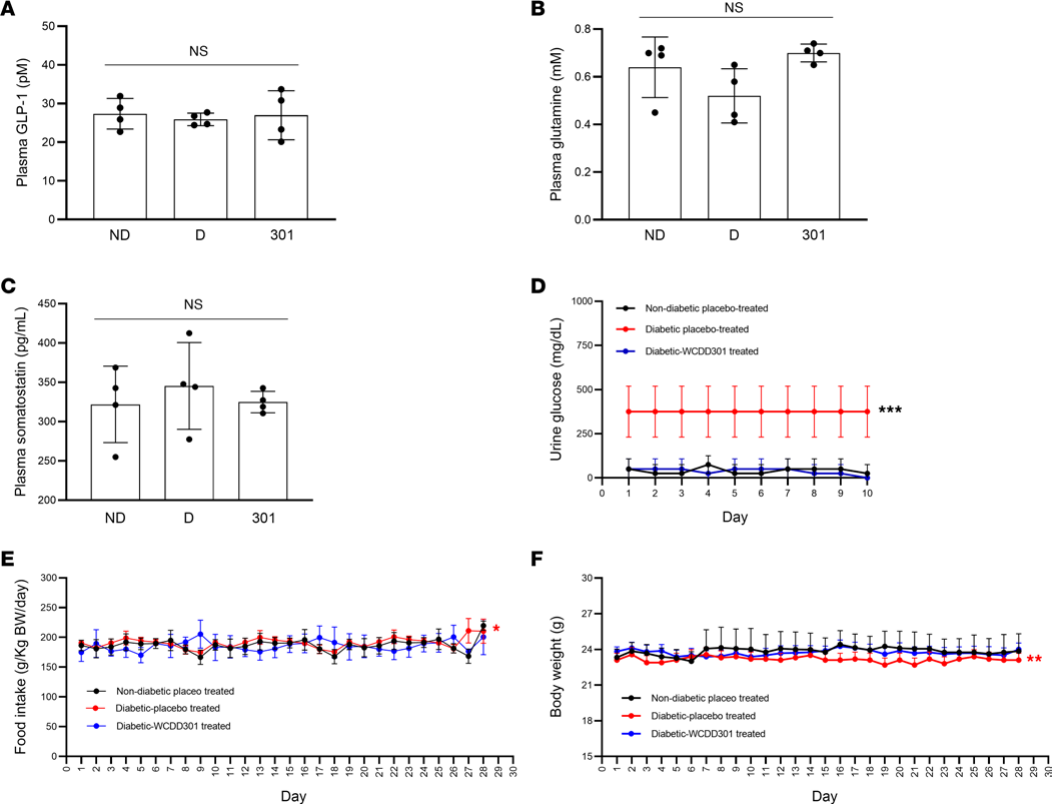
WCDD301 does not alter plasma levels of GLP-1, glutamine, and somatostatin and normalizes hyperglucosuria, polyphagia, and weight loss in diabetic NOD mice. Plasma levels of (**A**) GLP-1, (**B**) glutamine, and (**C**) somatostatin in nondiabetic placebo-treated (ND), diabetic placebo-treated (**D**), and diabetic WCDD301-treated NOD mice. Each dot represents mean values for 1 mouse. Values were compared among groups using 1-way ANOVA (*n* = 4). Daily (**D**) urine glucose, (**E**) food intake, and (**F**) body weight in nondiabetic placebo-treated, diabetic placebo-treated, and diabetic WCC301-treated NOD mice. Values (*n* = 4, mean ± SEM) of diabetic placebo-treated mice compared with the other groups; 1-way ANOVA, α = 0.05. *P < 0.05, **P < 0.01, ***P < 0.001.

WCDD301-associated reduction of blood glucose (Figure 6A) was accompanied by suppression of glucagon levels (Figure 6B) even in the presence of diminished blood insulin levels (Figure 6C). Glucose tolerance was improved in NOD mice treated with WCDD301 compared with the placebo-treated diabetic control (Figure 6D), and insulin tolerance in WCDD301-treated mice was similar to the placebo-treated diabetic controls (Figure 6E). WCDD301 also normalized hyperglycemia in streptozotocin-induced (STZ-induced) diabetic mice over a 4-week dosing regimen (Figure 6F). WCDD301 lowered plasma glucagon levels but did not alter glucagon-stimulated glucose output from primary mouse hepatocytes (Figure 6G), nor did it affect euglycemia in nondiabetic NOD mice (Supplemental Figure 6B). Further, administration of WCDD301 did not alter plasma levels of other glucose homeostatic regulators, as plasma levels of glucagon-like peptide 1 (GLP-1) (Figure 7A), glutamine (Figure 7B), and somatostatin (Figure 7C) were similar between WCDD301-treated diabetic NOD mice and nondiabetic placebo-treated control mice. Daily monitoring of urine glucose (Figure 7D), food intake, (Figure 7E), and body weight (Figure 7F) in diabetic WCDD301-treated mice also showed phenotypes similar to nondiabetic placebo-treated mice. Histology did not indicate any alterations in numbers or sizes of α cells in WCDD301-treated mice compared to either placebo-treated diabetic or nondiabetic mice (Supplemental Figure 7, A and B). WCDD301-treated animals appeared healthy and active, and blood chemistry markers for kidney function (blood urea nitrogen [BUN], creatinine), liver function (albumin [ALB], alanine aminotransferase [ALT]), and general pathology (aspartate aminotransferase [AST]) remained in the normal range (Supplemental Table 2). Taken together, these data point to reduced glucagon secretion as the primary action of WCDD301, with minimal or no toxicity over a 3-month administration regimen.

## Discussion

We developed a small molecule agonist of the EphA4 receptor to target suppression of glucagon secretion in α cells as a treatment of hyperglucagonemia-hyperglycemia in diabetes (32, 35). Our chemical design utilized knowledge of the ligand binding domain (LBD) of the EphA4 receptor (mouse EphA4: Uniprot Q03137, human EphA4: Uniprot P54764) combined with structural characteristics of known EphA4 agonists (36, 37, 40).

We synthesized a series of compounds that led to WCDD301, which was originally described via a 1-step synthesis in 1897 (41). The 1-step synthesis has a low efficiency (~6%), but our 3-step synthesis from commercial starting materials is readily scaled up for investigational new drug–enabling studies and clinical supply. WCDD301 shows a strong affinity (*K_i_* ≤ 0.13 μM) for the EphA4 receptor, comparable with other reported EphA4 agonists (40). In recent years, EphA4 agonists have been proposed for a variety of therapeutic applications, including amyotrophic lateral sclerosis (37) and prostate cancer (36).

To target this pathway for the treatment of hyperglycemia in T1D, whereas the available compounds used for other diseases were not effective in reducing glucagon hypersecretion from either mouse or human islets (Supplemental Figure 1, A–E), we showed a direct interaction between WCDD301 and the EphA4 receptor in vitro (Figure 1, B–E) and that this interaction recapitulates the action of soluble Ephrin-A5 in both isolated islets and dispersed islet cells. Our data further show suppression by WCDD301 of glucagon secretion from isolated islets and dispersed islet cells of patients with T1D (Figure 2, G–J). WCDD301 also blocks arginine-stimulated glucagon secretion (Supplemental Figure 2) but does not affect glucose release from mouse hepatocytes (Figure 6G). These actions appear to signal through EphA4 by the same RhoA signaling pathway as the receptor’s natural ephrin ligand, and both are accompanied by increased cortical F-actin (35).

Noncovalent drugs, including WCDD301 as a reversible competitive agonist (Figure 1, D, E, and H), generally show lower risks of unpredicted toxicity than covalent drugs (42). Our data are consistent with low toxicity both in vitro and in vivo. WCDD301 does not affect the secretory function of β cells over a range of concentrations (Figure 2, D–F), and WCDD301 administration achieves euglycemia without clinical signs of illness, hepatotoxicity, or nephrotoxicity (Supplemental Table 2). Blood chemistry assays after WCDD301 administration show normal levels of BUN, creatinine, ALB, and transaminases (AST, ALT) that are established markers for nephrotoxicity, hepatotoxicity, or multiple-organ injury (43). Interestingly, prophylactic administration of WCDD301 appears to prevent the development of diabetes in NOD mice (Supplemental Figure 5B), which is consistent with histology showing the prevention of mononuclear cell infiltration in islets of diabetic NOD mice (Figure 4C). This may suggest a role for WCDD301 similar to teplizumab in protecting β cells in people at risk for T1D (44, 45).

We have shown that WCDD301 agonizes the EphA4 receptor and suppresses glucagon hypersecretion from α cells (Figure 1F and Figure 2, A–C) accompanied by increased F-actin polymerization (Figure 3, A, B, E, and F, and Figure 4, D–H). The activated ephrin-EphA4 complex is expected to be internalized into cells to terminate signaling (46–50). This mechanism is consistent with our data showing increased immunostaining of the N-terminal sequence of EphA4 receptor within the α cell following treatment with WCDD301 (Figure 3, C, D, G, and H). This phenomenon is further consistent with our data showing reduced shedding of EphA4 into the cell culture medium following treatment with WCDD301 (Supplemental Figure 4D).

WCDD301 shows excellent stability in liver microsomes and plasma in both the human and mouse. Given the high clearance rates of hydrophilic compounds (51), which is in line with our intravenous pharmacokinetic findings (Supplemental Figure 5B), we formulated WCDD301 in a time-release form using SAIB-Agar excipient. This formulation normalized blood glucose within 2 weeks and maintained normalized levels during an 11-week follow-up (Figure 5A) in diabetic NOD mice with moderate hyperglycemia, consistent with early-stage diabetes in NOD mice (12, 52). Increased plasma duration of WCDD301 following subcutaneous administration (Supplemental Figure 5B) suggests that the oral sustained-release pearl provides a long plasma bioavailability. However, because of low plasma concentration following the time-release dosing, measuring WCDD301 plasma half-life following oral administration remains elusive.

We tested our proposed mechanism of EphA4 agonism by WCDD301 suppressing glucagon hypersecretion and normalizing hyperglycemia (53) with numerous control experiments. We did not observe any effects of WCDD301 on plasma levels of glucose homeostatic regulators (GLP-1, somatostatin, glutamine; Figure 7, A–C), α cell number/size (Supplemental Figure 7, A and B), glucosuria (Figure 7D), food intake (Figure 7E), body weight (Figure 7F), or peripheral insulin sensitivity (Figure 6E)). We also tested WCDD301 in various ex vivo models including intact isolated islets and dispersed islet cells from both humans and mice (Figure 2, G–J, and Figure 1, F and G). Dispersed islet cells approximate the T1D situation where α cells are separated from β cells, although this preparation does not recapitulate possible interactions from residual or malfunctioning β cells. This limitation is partially addressed by our validation that WCDD301 shows similar action on islets from human T1D donors (Figure 2, G and H). Our results are similar to another study of T1D treatment using a small molecule (SRI-37330) that exhibited reduced serum glucagon accompanied by increased insulin (19), and we cannot rule out a role for very low levels of insulin (54, 55). Further, the action of WCDD301 during challenging conditions, such as insulin-induced hypoglycemia (56) and dietary or nondietary glycemic volatility (57), remain to be elucidated.

In conclusion, we developed a small molecule drug, WCDD301, that agonizes the EphA4 receptor on α cells and suppresses glucagon hypersecretion. By formulating this water-soluble molecule with a hydrophobic excipient, we showed that oral delivery of this compound normalizes blood glucose in T1D mouse models. Taken together, these data suggest that the α cell EphA4 receptor is a druggable target for normalization of hyperglycemia in patients with T1D.

## Methods

### Synthesis of WCDD301

We used 2 synthetic approaches for development of 2-(acetoxymethyl)-2-aminopropane-1,3-diyl diacetate that we named WCDD301.

#### Method 1: small-scale synthesis.

As the chemical reaction in Supplemental Figure 8 shows, commercial tris (hydroxymethyl) aminomethane hydrochloride (500 mg, 3.2 mmol) was dissolved in a mixture of acetic anhydride (1.2 mL, 11.7 mmol): acetic acid (1.5 mL) and heated at 110°C for 5 hours. The solvent was removed in vacuo, and the residue was washed 3 times using diethyl ether. The residue was recrystallized from ethanol–diethyl ether to yield WCDD301 (53.5 mg, 6.2% yield, 100% purity).

#### Method 2: scale-up synthesis.

As the chemical reaction in Supplemental Figure 9 demonstrates, commercial compound 1 (5 g, 22.60 mmol, 1.90 mL, 1 eq) was dissolved in dichloromethane (DCM; 50 mL) followed by adding triethanolamine (9.15 g, 90.40 mmol, 12.58 mL, 4 eq) and acetyl chloride (5.32 g, 67.80 mmol, 4.84 mL, 3 eq) in a dropwise manner into the solution at 0°C under inert N_2_ atmosphere. The mixture was stirred at 20°C for 4 hours until completion of the reaction. When liquid chromatography-mass spectrometry (LC-MS) showed completion of the reaction, the resulting mixture was filtered and concentrated in a vacuum. This yielded compound 2 (7.5 g, 21.6 mmol, 95.6% yield) as a yellow oil. Trifluoroacetic acid (TFA; 20 mL) was added to a solution of compound 2 (7.5 g, 21.6 mmol, 1 eq) in DCM (60 mL). The mixture was stirred at 20°C for 2 hours. Completion of the reaction was monitored by LC-MS, and the mixture was concentrated in vacuo. The residue was purified by HPLC to obtain WCDD301 as a TFA salt (3 g, 8.31 mmol, 56.2% yield, 100% purity): 1HNMR (400 MHz): MeOD δ 4.32 (s, 6H), 2.14 (s, 9H); mass spec (M+H) + *m/z* 248.0.

### Measuring the binding of Ephrin-A5 and EphA4 receptor

Wells of the protein A–coated microplate (Pierce, catalog 15132) were washed using 200 μL of binding buffer (PBS supplemented by 0.01% Tween 20). Recombinant mouse EphA4 Fc chimera protein (R&D Systems, catalog 641-A4-200) was reconstituted in 1× PBS (Genesee, catalog 25-507XB, pH 7.2 ± 0.1), diluted to 3 μg/mL using binding buffer, dispensed (100 μL) in each well, and rocked on a belly dancer shaker (3 hours at room temperature, RT). Following 3 washes in the binding buffer, blocking was done using blocking buffer (5% BSA in binding buffer) for 3 hours at RT. Then, after 5 washes, the reconstituted recombinant mouse Ephrin-A5 Fc (R&D Systems, catalog 7396-EA-050) was added (100 μL) into each well and rocked for 2.5 hours on the shaker (Ephrin-A5 Fc concentrations were used as described in the Results section). After 7 washes using binding buffer, alkaline phosphatase–conjugated antibody against Ephrin-A5 was diluted (1:1,000) in the binding buffer plus 2% BSA, added to each well, and incubated at 4°C, overnight. Conjugation of the anti-EFNA5 antibody (Abnova, catalog H00001946-M02; 0.5 mg/mL; target sequence 114–203) was done using Abcam conjugation kit (catalog ab102850) based on the manufacturer’s instruction. After washing 7 times, *p-*nitrophenyl phosphate tablet (MilliporeSigma, catalog N1891) was reconstituted in 5 mL of PBS and added to each well (100 μL). Following 1 hour shaking at RT in the dark, reaction was stopped by adding 100 μL of 3N NaOH. OD was read at 405 nm within 5 minutes using the microplate reader (Agilent BioTek Cytation 5).

To determine binding affinity (*K_i_*) of WCDD301 for EphA4, in the designed ELISA system, Ephrin-A5 Fc was incubated in EphA4-coated wells in the presence or absence of WCDD301 (0.1 μM). As a control, Fc fragment (BioLegend, catalog 790102) was incubated and used for normalization of data. By running the experiment at a range of Ephrin-A5 Fc (2, 4, 8, 16, 32, 64 nM), *K_m_* (*K_i_*) values were determined in the absence (*K_m_*) and presence of WCDD301 (*K_m_* obs). Then, *K_i_* was determined via an approach called finding *K_i_* without global fitting (58) by using the following formula:


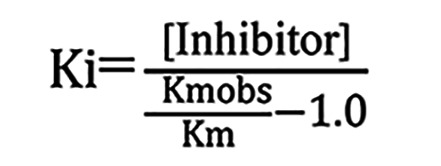
.

This approach provided a *K_m_* value for WCDD301 and an estimate for its binding affinity, *K_i_*. To further verify this finding, experiments were designed in the context of 8 nM Ephrin-A5 to determine bound levels of Ephrin-A5 in the presence of WCDD301 (0, 0.1, 0.4, 0.8, and 1.6 μM). Data were normalized and fit by nonlinear regression in GraphPad Prism software version 4 to determine IC_50_. Then, by using the Cheng-Prusoff equation


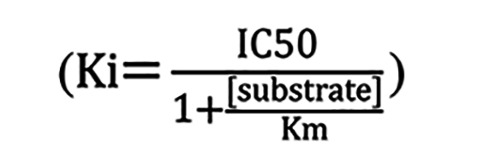


(59), a *K_i_* value was determined.

### Determining competition between WCDD301 and Ephrin-A5 for binding to EphA4 receptor

We took following the approaches to assay competition between Ephrin-A5 and WCDD301 for binding to the EphA4 receptor. Wells of the microplate were coated by a fixed level of EphA4 Fc (3.5 pmol/well), and competition experiments with Ephrin-A5 and WCDD301 were performed as listed in Supplemental Tables 3 and 4. We determined the *K_D_* value for each condition by means of Michaelis-Menten best fit analysis in GraphPad Prism software version 4. To control for potential interaction between WCDD301 and Ephrin-A5 Fc, we coated wells of a microplate using Ephrin-A5 Fc and then incubated them with or without WCDD301 (0.2 μM).

### Islet cell dispersion

Pancreata of nondiabetic C57BL/6J mice were digested using collagenase P (Roche Diagnostics, catalog 11213873001), and islets were manually picked up using a stereomicroscope (ZEISS Stemi 305). Following an overnight resting in RPMI 1640 (Gibco, catalog 11879-020) supplemented with 11 mM glucose and 10% FBS, islets were subjected to dispersion. Isolated mouse islets or provided human islets were dispersed as described (28). Briefly, 100–120 islets were washed 2 times using modified HBSS, then dispersed in 600 μL Accutase (Innovative Cell Technologies, catalog AT-104) by gently pipetting up and down for 7 minutes at 37°C. Following neutralization of enzyme using supplemented RPMI 1640 (10% FCS, 10 mM HEPES, and 1% penicillin-streptomycin), cells were washed (2 times using HBSS containing 11 mM glucose) and rested in KRBH buffer (115 mM NaCl; 4.7 mM KCl; 2.6 mM CaCl_2_; 1.2 mM KH_2_PO_4_; 1.2 mM MgSO_4_; 10 mM HEPES; 5 mM NaHCO_3_; 0.1% BSA; 11 mM glucose, pH 7.4) for 1 hour. Following refreshing of KRBH, cells were dispensed in 8 microfuge tubes (2,000 cells/tube) and treated with WCDD301, preclustered Ephrin-A5 Fc, or preclustered Fc in the presence or absence of the competitive inhibitor of EphA4 receptor, rhyncophylline (60) (MilliporeSigma, catalog SML2345). In the presence or absence of these effectors, secretion assay was performed sequentially as shown in Supplemental Table 5. We also tested the effect of a reported potential agonist of EphA4, doxazosin (61) (TCI, catalog D4126), or its conjugate derivatives with lysine, tyrosine, or proline on glucagon secretion from dispersed islet cells. Preclustering of Ephrin-A5 Fc was done by mixing Ephrin-A5 Fc or Fc alone (BioLegend, catalog 790102) with anti-Fc antibody (Invitrogen, catalog A16086) (1:10 molar ratio) 20 minutes before treatment (62). Glucagon secretion was measured (Promega, Lumit Immunoassay), and its reduction was considered a reflective measure for binding of WCDD301 with LBD of EphA4 receptor. Shedding of cleaved EphA4 was measured from the cell culture medium. Experiments were performed by dispersion of 200 islets, and EphA4 levels in media were measured by EphA4 ELISA (ABclonal, catalog RK06572).

### Primary mouse hepatocyte culture

Primary hepatocyte culture was performed following previous publications (63, 64). Briefly, livers were extracted from C57BL/6J (*n* = 5), rinsed in HBSS, and minced. The minced tissue was treated with collagenase P solution (9 μg/mL) neutralized by DMEM containing 10% FBS. Following settling of undigested tissue, the supernatant was collected and centrifuged at 490*g* for 1 minute. The pellet was treated with red blood cell lysis buffer (8.26 g NH_4_Cl, 1 g NaHCO_3_, 0.037 g Na_2_EDTA in 1,000 mL of distilled water) for 3 minutes, diluted with HBSS containing 6% FBS, and centrifuged at 490*g* for 1 minute. The resulting pellet was washed 2 times using HBSS containing 6% FBS (290*g*, 1 minute, and 190*g*, 1 minute) and resuspended in HBSS containing 2% FBS. The procedure was repeated 2 additional times on the undigested tissue. The collected suspension was sequentially filtered through a 500 μm Steel Basket-Strainer (pluriSelect) and Falcon 100 µm Cell Strainer (Corning). After centrifugation (190*g*, 1 minute), the pellet was resuspended in DMEM (containing 10% FBS, 100 U/mL penicillin, and 100 μg/mL streptomycin), seeded in a 48-well cell culture plate (7.5 × 10^5^ cells/ well), and incubated in a cell culture incubator, overnight. Cell viability and attachment were determined by trypan blue staining. Following wash in PBS (3 times), cells were preincubated in glucose production medium (DMEM; Gibco, catalog 14430-01, supplemented with 2 mM sodium pyruvate, 20 mM sodium lactate, and 4 mM l-glutamine) for 2 hours. Then, culturing was continued for 6 hours in the presence or absence of glucagon and/or WCDD301. Media were collected for fluorometric glucose assay (Abcam, catalog ab169559), and cells were lysed in lysis buffer (150 mM NaCl and 1% Triton X-100 in 50 mM Tris-HCl, pH 7.4) for protein measurement (Thermo Fisher Scientific, catalog 23235).

### Human donor islet and dispersed islet cell experiments

The Integrated Islet Distribution Program provided us human donor islets with their characteristics (Supplemental Table 6). Islets were centrifuged at 500*g* for 3 minutes, resuspended in CMRL 1066 medium (Gibco, catalog 1150-037) containing 11 mM glucose, and rested in an incubator overnight.

For the hormone secretion assay, islets or dispersed islet cells were adapted in KRBH buffer containing 11 mM glucose for 1 hour. Then, after 2 washes in HBSS containing 11 mM glucose, islets or cells were treated in the following sequential order in the presence or absence of WCDD301: incubation in KRBH (1 mM glucose, 30 minutes) followed by medium collection, incubation in KRBH (11 mM glucose, 30 minutes) followed by medium collection. We measured concentrations of glucagon, insulin (Promega, Lumit Immunoassay), and somatostatin (Phoenix Pharmaceuticals, Chemiluminescent Enzyme Immunoassay) in the collected media, then compared the values among groups using 1-way ANOVA (α = 0.05).

Glucagon and insulin levels in buffer medium were measured by Lumit assay (Promega). Plasma concentrations of glucagon and insulin were determined by ELISA (8, 65).

### Immunofluorescence imaging of cells and pancreatic slices

Dispersed islet cells of healthy humans (*n* = 3) or mice (*n* = 5) were seeded on coverslips coated with 5 μg/mL rhLaminin-521 (Gibco, catalog A29249) and incubated (24 hours) in RPMI 1640 supplemented by 11 mM glucose, 10% FCS, 10 mM HEPES, and 1% penicillin-streptomycin in cell culture incubator. Coverslips were treated with WCDD301, preclustered Ephrin-A5 Fc, or preclustered Fc in the following sequential order: KRBH (11 mM glucose, 30 minutes), KRBH (1 mM glucose, 60 minutes), KRBH (1 mM glucose, 30 minutes), and KRBH (11 mM glucose, 60 minutes). After treatment, coverslips were washed once in PBS, fixed in 2% paraformaldehyde in PBS (30 minutes), and again washed in PBS. After 1-hour incubation in blocking buffer (2% BSA in PBS containing 0.1% Tween 20), coverslips were incubated with primary antibodies against glucagon (R&D Systems, catalog MAB1249, 1:100) and EphA4 (ABclonal, catalog A8346, 1:100; targeting the extracellular domain of EphA4, aa 20–100) at 4°C in a wet chamber, overnight. Following wash in PBS, coverslips were incubated in a mixture of Alexa Fluor 555 Phalloidin (Cell Signaling Technology, catalog 8953) and secondary antibodies (goat anti-rabbit Alexa Fluor 488, catalog A-21094, 1:1,000, Molecular Probes; and goat anti-mouse Alexa Fluor 633, catalog A21052, 1:1,000, Life Technologies) for 1 hour at RT. Both primary and secondary antibodies were diluted in blocking buffer containing 0.05% Tween 20. Coverslips were washed using PBS and counterstained by using DAPI (1:1,000) for 10 minutes. Following 2 washes in PBS, coverslips were mounted on glass slides using ProLong Glass Antifade Mountant (Invitrogen, catalog P36980). Images were captured using the Zeiss LSM880 microscope with a Plan-Apochromat 63× 1.4 NA objective lens by setting dual-band dichroic/filter for 405, 488, 561, and 633 nm laser excitation. For each slide, 25–30 fields of view were scanned, and in each field of view, 3–5 cells were imaged. For analysis, a region of interest (ROI) was manually drawn around each glucagon^+^ cell, and the intensity of F-actin or EphA4 within the ROI was determined using the intensity algorithm of ImageJ software (NIH). Values of intensity were compared among groups using 1-way ANOVA, α = 0.05.

Pancreata of NOD mice (females, *n* = 3 per group) from nondiabetic, diabetic placebo-treated, and diabetic WCDD301-treated cohorts were excised; fixed in 10% buffered formalin for 5 days; and treated with ethanol 70% for 2 days before paraffin embedding. The paraffin-embedded tissue blocks were longitudinally sectioned in 5 μm slices and fixed on microscope slides. Following deparaffinization (through incubation in graded xylene, graded ethanol, and PBS), permeabilization (using 0.1% Triton X-100 in PBS), and background blocking (using 5% BSA solution containing 0.1% Tween 20), samples were incubated with primary antibodies (mouse anti-glucagon, Abcam, catalog ab10988; above-mentioned rabbit anti-EphA4), secondary antibodies (goat anti-mouse IgG Alexa Fluor 488; Invitrogen, catalog A-11001; goat anti-rabbit IgG Alexa Fluor 633, Invitrogen, catalog A-21070), SPY555-Actin (Cytoskeleton, catalog SC202), and DAPI. Image acquisition and analysis were performed as for dispersed islet cells. To determine numbers of α cells per islet, intensity of glucagon signal/μm^2^ of islet was determined as a reflective measure of α cell numbers per islet. ROIs were manually drawn around each islet, background signal was removed by thresholding, and intensity of glucagon signal was determined and normalized by the islet surface area. To determine sizes of α cells per islet, ROIs were manually drawn around α cells in each islet. Paraffin-embedding, sectioning of the embedded blocks, and Hematoxylin-Eosin staining were performed by the Anatomic and Molecular Pathology Core Labs, Washington University in St. Louis.

### Plasma stability assay of WCDD301

Pooled frozen plasma of CD-1 mice (*n* = 20; catalog MSE00PLK2M2N, Beijing Vital River Laboratory Animal Technology) or humans (*n* = 6, catalog HUMANPLK2P2N, Bioreclamation IVT), equal numbers of males and females, was thawed in a 37°C water bath, and residual clots were removed by centrifugation (500*g* for 5 minutes). Plasma (98 μL) was aliquoted in each well of 96-well reaction plates using an Apricot automation workstation. WCDD301 (100 μM) or propantheline bromide (100 μM, as a positive control) was added (2 μL/well) to sets of wells assigned to blank or time points (T_0_, T_10_, T_30_, T_60_, and T_120_ minutes) and incubated at 37°C. At each time point, the reaction was stopped by adding stop solution (500 μL; a mixture of 200 ng/mL of tolbutamide and labetalol in acetonitrile), mixed thoroughly, sealed, shaken (20 minutes), and centrifuged (1,750*g*, 4°C, 20 minutes). A total of 150 μL of the supernatant from each well of the reaction plate was transferred to its corresponding bioanalysis plate for LC-MS/MS analysis. The remaining level (%) of the WCDD301 was calculated using the following equation: remaining (%) = (PAR at each time point/PAR at T_0_) × 100. PAR stands for the peak area ratio of the WCDD301 versus internal standard.

### Microsomal stability assay of WCDD301

Liver microsomes (human, Corning, catalog 452117; CD-1 mice, Xenotech, catalog M1000) were diluted to 0.56 mg/mL in 100 mM PBS as a working solution. A total of 445 μL of the working solution was transferred into prewarmed incubation plates for treated (T60) and control (NCF60) measurements. After preincubation (10 minutes at 37°C with constant shaking), 54 μL was transferred to the blank plate, followed by 6 μL of NADPH and 180 μL quenching solution. In parallel, 5 μL of WCDD301 (100 μM) was added into incubation plates (T60 and NCF60) containing microsomes and mixed thoroughly (3 times). For the NCF60 plate, 50 μL of buffer was added, mixed (thoroughly, 3 times), shaken, and incubated at 37°C for 60 minutes. In the quenching plate T0, quenching solution (180 μL) and NADPH cofactor (6 μL) were added. For T60 plate, 54 μL of the mixture was placed into the quenching plate (for T0 point), followed by adding 44 μL NADPH cofactor with shaking incubation at 37°C for 60 minutes. Quenching solution (180 μL) was added to quenching plates at 5, 15, 30, 45, and 60 minutes, and 60 μL of sample was transferred from T60 plate per time point to the quenching plates. For the NCF60 plates, 60 μL sample from the NCF60 incubation plate was transferred to the quenching plate (containing quenching solution) at the 60-minute time point. After plates were shaken for 10 minutes and centrifuged (1,750g, 20 minutes at 4°C), 80 μL of the supernatant was transferred into 240 μL HPLC water for LC-MS/MS analysis. The following first-order kinetic equation was used to calculate half-life (T1/2): 
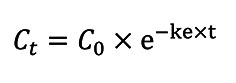
; when


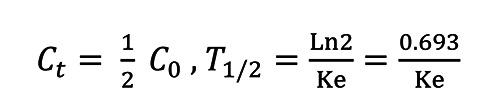
.

Then, intrinsic clearance [CLint (microsomal; mic)] and hepatic clearance [CLint (liver)] were predicted using the following equations (66):


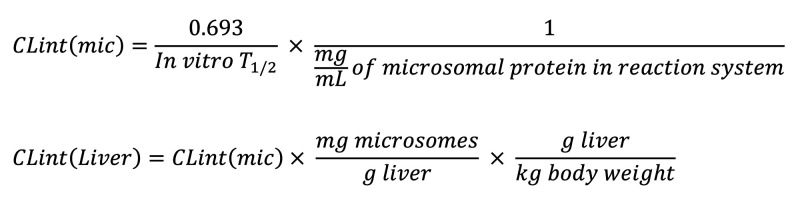


Pharmacokinetics

CD-1 mice were injected with WCDD301 subcutaneously (1 mg/kg, *n* = 3) or intravenously (1 mg/kg, *n* = 3), and blood was collected from saphenous vein directly into K_2_-EDTA–containing microcentrifuge tubes at 5, 10, and 15 minutes postdosing. An aliquot of 20 μL plasma was deproteinized using 200 μL of internal standard solution (100 ng/mL of labetalol and tolbutamide in acetonitrile), incubated for 10 minutes at RT, and centrifuged at 1,800*g* for 10 minutes at 4°C. Following 1:1 dilution of the supernatant with deionized water, 1 μL of the diluted sample was used for LC-MS/MS analysis (TripleQuad 6500, column: ACQUITY UPLC BEH C18 2.1 × 50 mm, 1.7 μm; flow rate: 0.6 mL/min, retention time: 1.60/1.78 WCDD301/labetalol).

### Animal models

Mice were purchased from the Jackson Laboratory and kept on a 12-hour light/12-hour dark cycle in the animal care facility at Washington University (St. Louis, Missouri, USA, Association for Assessment and Accreditation of Laboratory Animal Care International [AAALAC] accreditation D16-00245). Mice had access to water and regular chow diet ad libitum. All animal procedures were reviewed and approved by the institutional animal care and use committee (IACUC).

#### NOD/shiLTJ diabetic mice.

For the polygenic model for T1D (the Jackson Laboratory), 10 cohorts of independent NOD/shiLTJ mice (each 10, equal numbers of females and males) were purchased at 7–9 weeks old. Blood glucose levels were monitored twice a week using a glucometer (Glucocard Vital) to find those mice at risk of diabetes. Diabetes was confirmed when the blood glucose levels remained at 200–250 mg/dL for 3 consecutive days. This profile of the blood glucose level is referred to as moderate hyperglycemia.

#### STZ-induced diabetic mice.

Six cohorts of C57BL/6 mice (*n* = 10 each, equal numbers of females and males; the Jackson Laboratory) were fasted for 5 hours and injected intraperitoneally with 40 mg/kg STZ solution (MilliporeSigma, catalog 572201) for 5 consecutive days. STZ solution (40 mg/mL) was prepared within 5 minutes before injection using freshly prepared citrate buffer (0.1 M, pH 4.5). Nine days after the last injection, blood glucose levels were measured for 2 consecutive days, and those animals with constant values of 300 ± 20 mg/dL were considered as having early severe hyperglycemia.

### Oral administration of WCDD301 in diabetic mice

We prepared sustained time-release pearls for oral administration in the mouse models. To this end, we combined FDA-approved excipients of Eastman BioSustane SAIB NF (product code: 41271-00, sample distribution from Eastman Company, Kingsport, Tennessee, USA; provided by Phillip Cook) and Agar (Lamda Biotech, catalog C110) at the ratio of 1:1.45 (w/w). We also prepared another type of sustained time-release pearl by mixing SAIB, Agar, and L (-) malic acid (MilliporeSigma, catalog 202-601-5) at the ratio of 1:1.4:0.2 (w/w/w). A homogenous paste mixture was prepared, and pearls were formed with 50–60 mg weight and approximately 2 mm diameter by pinching off the mixture and manually rolling.

We preweighed WCDD301 into microfuge tubes and reconstituted it using deionized water. Using a 10 μL pipette tip, we placed the calculated dose of WCDD301 (at the volume range of 1–1.5 μL) within the center of pearls. WCDD301 was placed into pearls 30–45 minutes before administration. For oral administration of pearls, we designed an applicator using a disposable plastic transfer pipette and syringe plunger lubricated with olive oil. Food intake and body weight in each experimental group (diabetic WCDD301-treated, diabetic placebo-treated, and nondiabetic placebo-treated NOD mice) were monitored daily for 4 weeks. Urine glucose was measured from individually caged NOD mice (nondiabetic placebo treated *n* = 2, diabetic placebo treated *n* = 2, and diabetic WCDD301 treated *n* = 4, 21–23 weeks old) with metabolic cages. Urine glucose was monitored every 24 hours for 10 days by semiquantitative measurements (P-STICKS, Millpledge Veterinary).

We applied the following therapeutic regimens in the fed state for oral dosing of pearls containing WCDD301. The therapeutic regimen for diabetic NOD mice with moderate hyperglycemia (*n* = 6–9; 6 females/3 males) or nondiabetic ones with blood glucose levels of < 140 mg/dL (females, *n* = 5) was dose 7.5 mg/kg, dosage once a day, and excipient SAIB-Agar, 50–60 mg. The therapeutic regimen for STZ-induced diabetic mice with early severe hyperglycemia (*n* = 3, placebo group; *n* = 4 treatment group, females) was dose 10 mg/kg, dosage once a day, and excipient SAIB-Agar-malic acid, 50–60 mg.

### Profiling blood chemistry parameters

Terminal blood was collected into microfuge tubes containing K_2_-EDTA (supplemented by 45 mM phenylmethylsulphonyl fluoride and 0.77 μM aprotinin) through cardiac puncture. Plasma was separated by centrifugation (2,000*g* for 10 minutes at 4°C) and kept at –80°C until analysis. Plasma samples with hemolysis were excluded from the study. Levels of plasma glucagon (Crystal Chem, ELISA kit, catalog 81520), insulin (Crystal Chem, ELISA kit, catalog 90080), GLP-1 (Crystal Chem, ELISA kit, catalog 81508), glutamine (MilliporeSigma, colorimetric, catalog MAK438), and somatostatin (Phoenix Pharmaceuticals, Chemiluminescent EIA kit, catalog CEK-060-03) were determined by ELISA. Values of BUN, creatinine, ALB, ALT, and AST were determined by a Roche Cobas C501 Autoanalyzer as performed by the Washington University Diabetes Research Center Translational Diagnostics Core. Reference intervals of blood chemistry parameters for inbred NOD mice were extracted from the Mouse Phenome Database (https://phenome.jax.org/).

### Glucose tolerance test

Following 5 hours of fasting, NOD mice were injected with 20% sterile glucose solution (i.p. 2 mg/kg body weight; at the volume of 10 times of the body weight). Blood glucose levels were measured before injection by tail nick. Following injection, blood glucose levels were measured at time points of 15, 30, 45, 60, 90, 120, and 180 minutes by using the glucometer. When blood glucose levels reached above 600 mg/dL, the glucometer showed HI, and we assigned a value of 650 mg/dL for this condition. Glucose tolerance test was done at 3 time points (8, 14, and 24 hours) after the final WCDD301 administration. Data were plotted using GraphPad Prism software version 4.

### Insulin tolerance test

Following a 4-hour fast, nondiabetic placebo-treated (*n* = 4), diabetic placebo-treated (*n* = 4), and diabetic WCDD301-treated (*n* = 4) NOD female mice were intraperitoneally injected with insulin (Humulin-R; 0.75 U/kg, Eli Lilly and Company). Blood glucose was measured immediately before injection (time 0) and at time points of 15, 30, 45, 60, and 120 minutes postinjection through tail snipping and an Arkray Glucocard Vital Blood Glucose Meter.

### Statistics

Comparison of values for treatment groups versus corresponding controls or other treatment groups was performed using 1-way ANOVA and post hoc tests of Dunnett at α = 0.05. Unpaired 2-tailed *t* test was used to compare values between 2 conditions. *P* < 0.05 was considered statistically significant.

### Study approval

All animal procedures were reviewed and approved by the IACUC, Washington University, St. Louis, Missouri, USA, AAALAC accreditation D16-00245.

### Data availability

All data are available in the main text, supplement, or Supporting Data Values XLS file.

## Author contributions

FA and DWP conceptualized the project and the chemical compound. FA synthesized the compound in primary form and tested it in vitro and ex vivo. RED synthesized the purified form of the molecule and performed the pharmacokinetics experiments. FA and SCG performed in vitro, ex vivo, and in vivo experiments. FA, DWP, and SCG performed the data analysis. All authors participated in writing the manuscript and approved the final version.

## Supplementary Material

Supplemental data

Supporting data values

## Version 1 01/23/2024

Electronic publication
