# Supplementary material for: An orally available compound suppresses glucagon hypersecretion and normalizes hyperglycemia in type 1 diabetes
Source: JCI Insight. 2024 Jan 23;9(2):e172626. doi: 10.1172/jci.insight.172626 (PMC10906223; doi:10.1172/jci.insight.172626)
Supplement: Supplemental data [file jciinsight-9-172626-s088.pdf]

Supplemental materials

Supplemental Figure 1

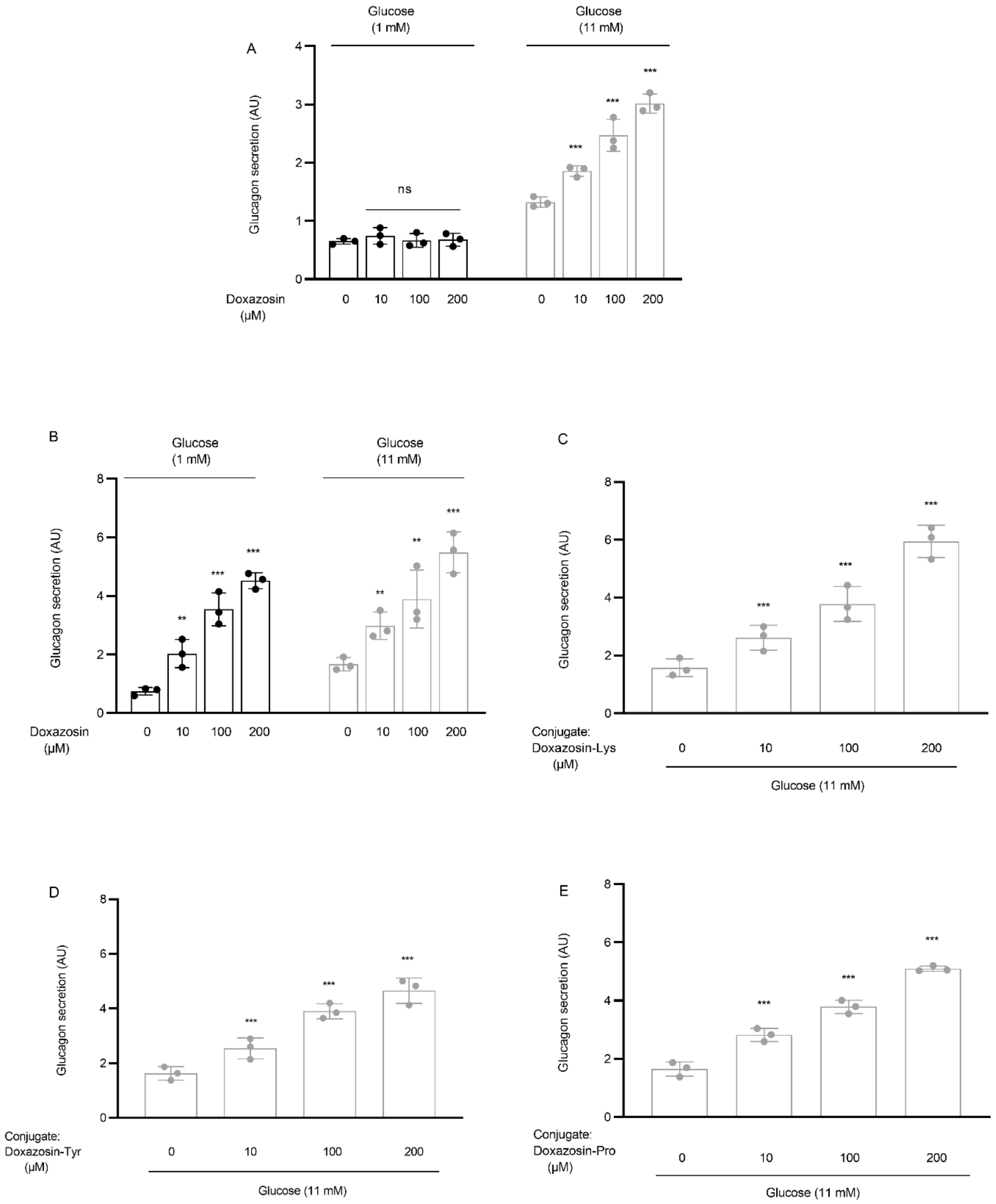

Supplemental Figure 1. Doxazosin and its derivatives compromise glucagon secretion in α-cells. Glucagon secretion from dispersed islet cells of (**A**) human and (**B**) mouse following treatment with Doxazosin. Glucagon secretion from mouse dispersed islet cells following treatment with conjugate of Doxazosin with (**C**) lysine (Doxazosin-Lys), (**D**) tyrosine (Doxazosin-Tyr) and (**E**) proline (Doxazosin-Pro). Each dot represents mean values for dispersed islet cells of one mouse or subject. Values (n=3, mean± SEM) were compared to the respective 0 μM control; 1-Way ANOVA; $^{**}p<0.01$, $^{***}p<0.001$.

Supplemental Figure 2

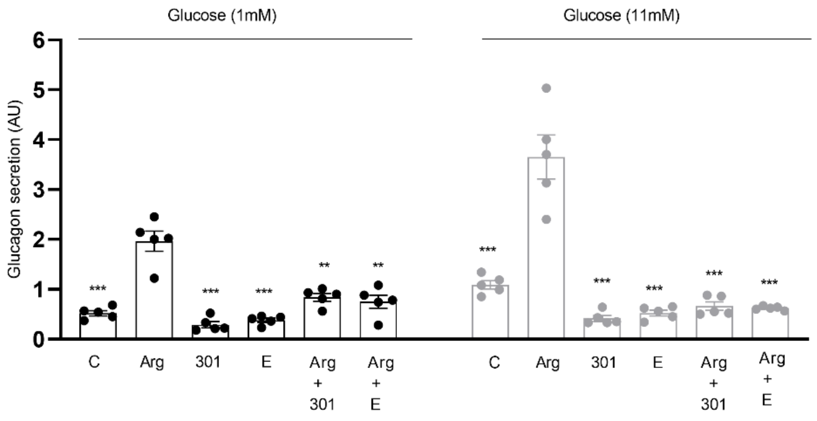

Supplemental Figure 2: WCDD301 blocks Arginine-stimulated glucagon secretion in murine dispersed islet cells. Glucagon secretion in dispersed murine islet cells in response to Arginine (Arg; 25 mM), WCDD301 (301; 3μM), Ephrin-A5 (E; 4 μg/mL), Arginine plus WCDD301 (Arg + 301), and Arginine plus Ephrin-A5 (Arg + E). Each dot represents values for one mouse. Values of each group (n=5, mean± SEM) compared to the Arginine stimulated group (Arg); 1-Way ANOVA. **p<0.01, ***p<0.001.

Supplemental Figure 3

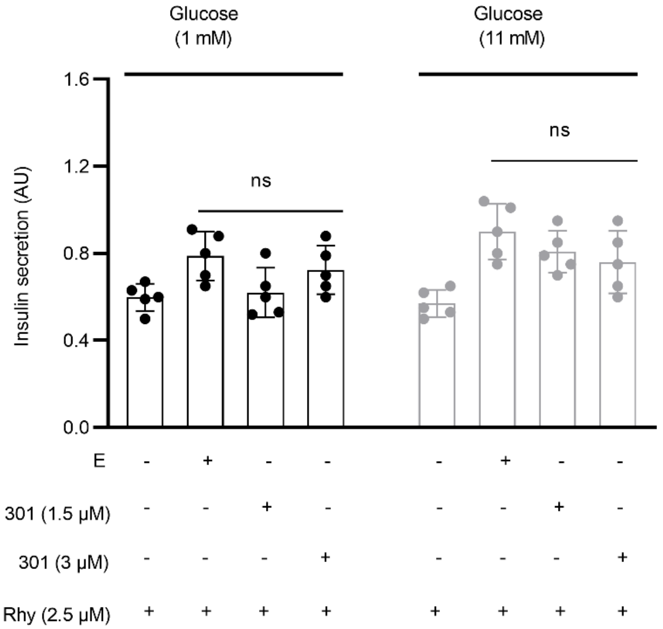

Supplemental Figure 3. WCDD301 and Ephrin-A5 do not modulate insulin secretion. Insulin secretion in dispersed murine islet cells in the presence and absence of EphA4 inhibitor (Rhy: Rhyncophylline), WCDD301 (301) or Ephrin-A5 Fc (E). Each dot represents values for one mouse. Values of each group (n=5, mean± SEM) compared to the respective control; 1-Way ANOA; ns (not significant).

Supplemental Figure 4

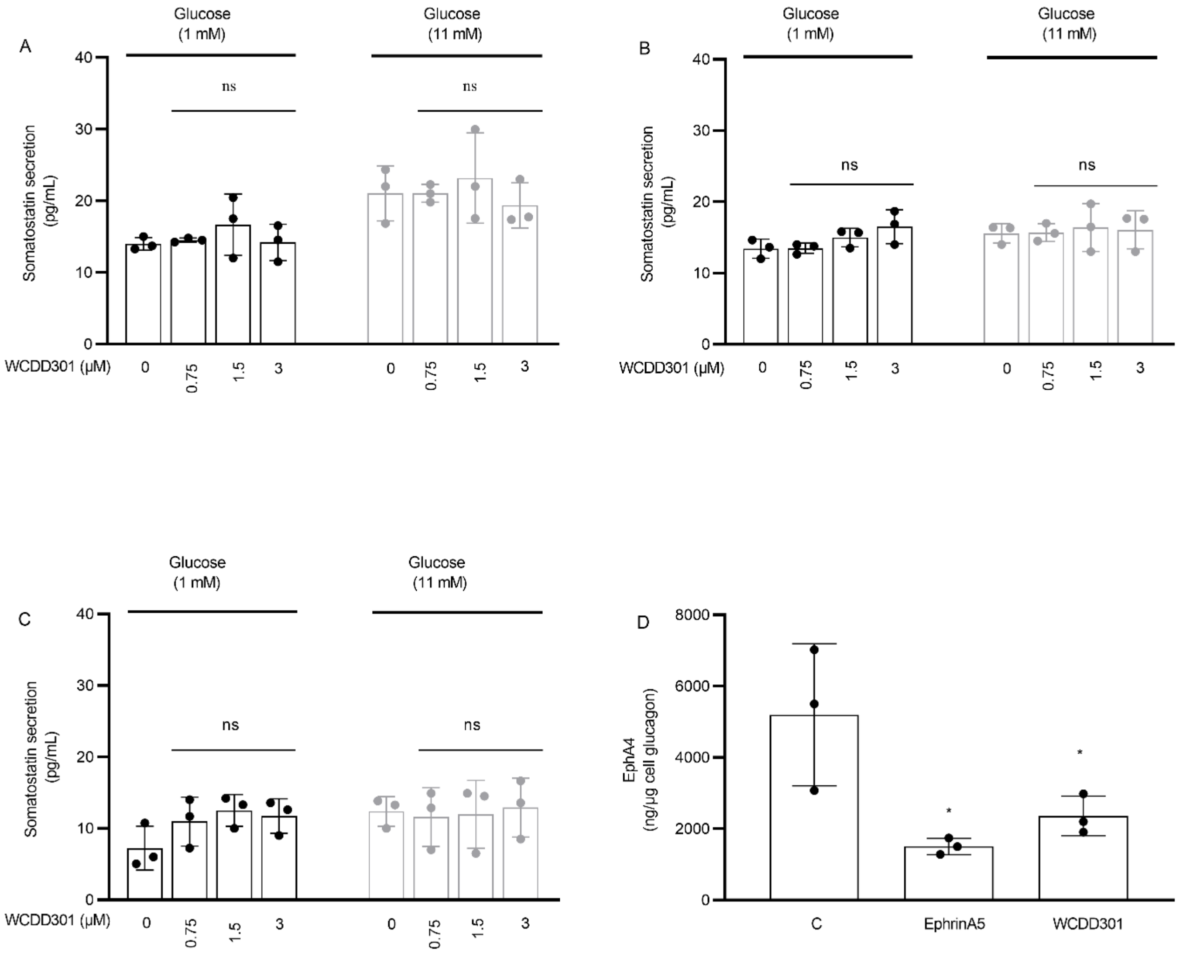

Supplemental Figure 4. WCDD301 does not compromise somatostatin secretion and lessen EphA4 shedding with no effect on glucose output from hepatocyte. Somatostatin secretion in non-diabetic human donor dispersed islet cells following (**A**) 1h, (**B**) 3h, or (**C**) 6h incubation with WCDD301. (**D**) EphA4 shed from dispersed mouse islet cells into culture medium of vehicle-treated (C) and treated groups with Ephrin-A5 Fc and WCDD301(301). Each dot represents mean values for dispersed islet cells of one subject or mouse. Values (n=3, mean± SEM) compared to the respective controls;1-Way ANOVA. *$p<0.05$; ns (not significant).

Supplemental Figure 5

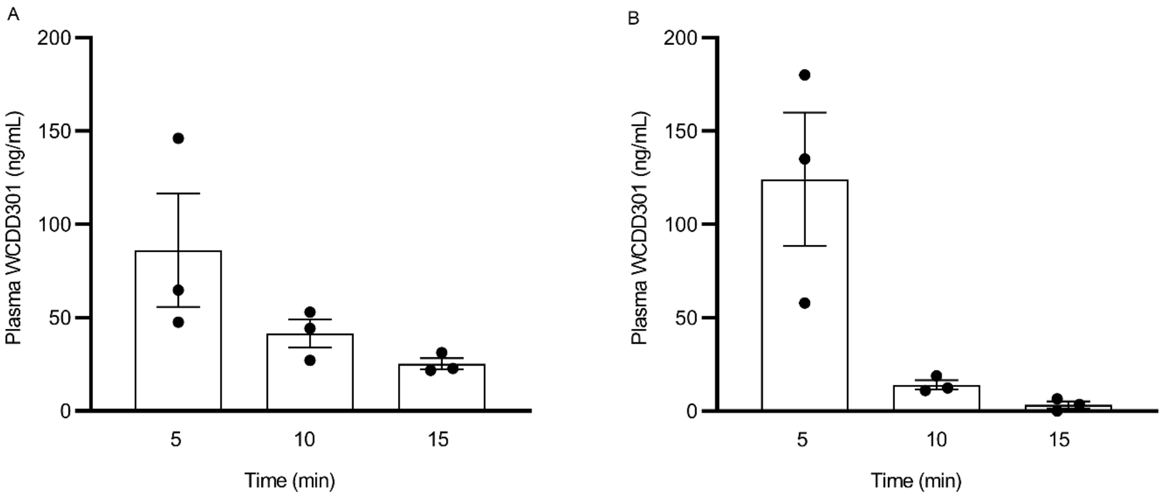

Supplemental Figure 5: Slow release of WCDD301 into bloodstream increases its plasma survival rate. (A) Subcutaneous (1mg/kg) and (B) Intravenous (1mg/kg) injections of WCDD301 in mice (n=3) and its plasma levels at 5. 10, and 15-min post-dosing. Each dot represents mean values in one mouse.

Supplemental Figure 6

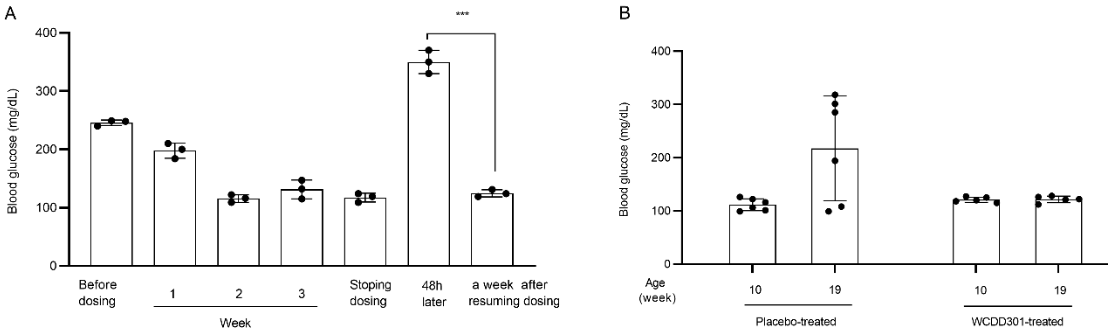

Supplemental Figure 6. Stopping WCDD301 dosing causes hyperglycemia and resuming dosing recovers normoglycemia in diabetic NOD mice; Normoglycemic NOD mice under WCDD301 dosing do not show phase transition towards hyperglycemia. (A) Stopping dosing of WCDD301 in treated diabetic NOD mice with WCDD301 and resuming dosing 48h later. Each dot represents mean values for one mouse. Comparison of values (n=3, mean ± SEM) was performed using unpaired t-test ***p<0.001. **(B)** Blood glucose levels at age 10 and 19 weeks in placebo-treated NOD mice (n=6) versus WCDD301- treated ones (n=5).

Supplemental Figure 7

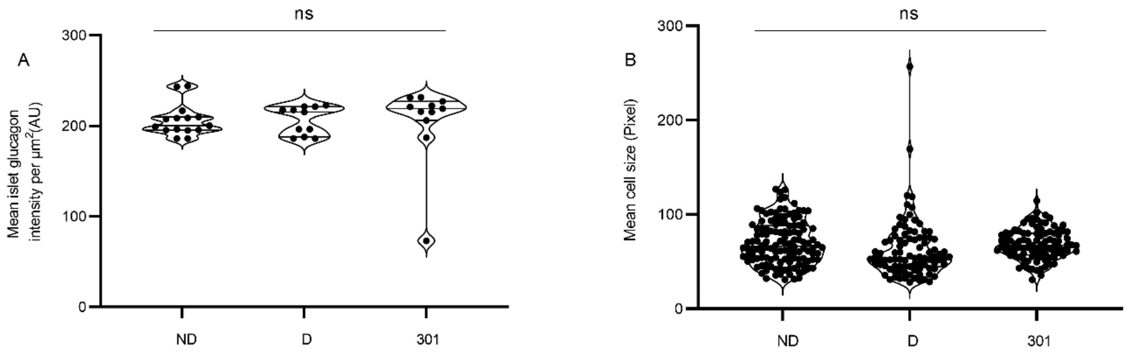

Supplemental Figure 7. WCDD301 dosing in NOD mice does not alter α-cell numbers or sizes. α-cell (**A**) numbers and **(B)** sizes following 11-week administration of WCDD301 or placebo in mice. In graph A, each dot represents threshold intensity of glucagon signals in each islet (n=11-15). In graph B, each dot represents α-cell areas in islets (n=84-97). Values (mean±SEM) compared among groups using 1-Way ANOVA; ns (not significant).

Supplemental Figure 8.

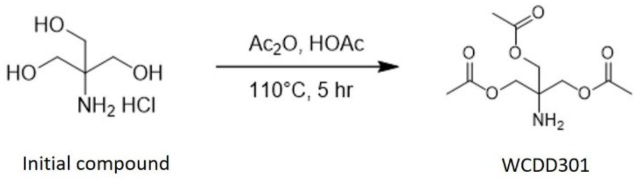

Supplemental Figure 8. One step synthesis of WCDD301 has a low production output. Chemical reaction for small scale synthesis of WCDD301 from the initial compound of tris (hydroxymethyl) aminomethane hydrochloride in the presence of Acetic anhydride (Ac2O) and Acetic acid (HOAc).

Supplemental Figure 9

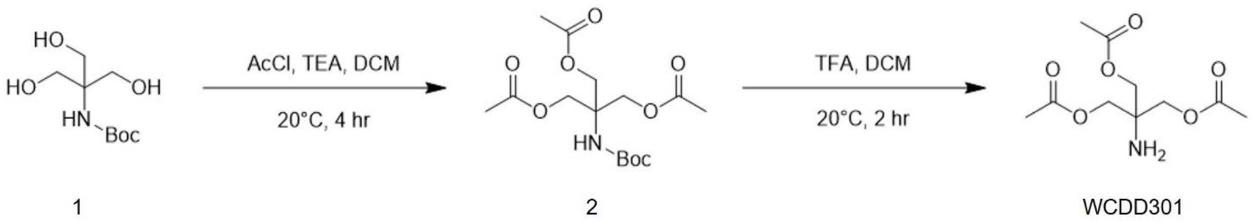

Supplemental Figure 9. Three-step synthesis of WCDD301 has a high production output. Chemical reaction for scale-up synthesis of WCDD301 following amine protection of the tris (hydroxymethyl) aminomethane hydrochloride using tert-Butyloxy carbonyl (Boc) and continuation of reactions in the presence of Acetyl chloride (AcCl), Triethylamine (TEA), Dichloromethane (DCM) and Trifluoroacetic acid (TFA).

Supplemental Table 1

Drug and controls	Plasma half-life (min)		Microsomal half-life (min)		Intrinsic microsomal clearance (µL/min/mg)		Hepatic clearance (mL/min/kg)	
	mouse	human	mouse	human	mouse	human	mouse	human
WCDD301	105.1	289.1	93.6	>145	14.8	<9.6	58.7	<8.6
Propantheline	34.3	9.8	-	-	-	-	-	-
Diclofenac	-	-	47.5	4.4	29.2	313.9	115.5	282.5
Propafenone	-	-	2.7	5.3	516.8	262.5	2046.6	236.3
Testosterone	-	-	5.4	14.5	257.2	95.6	1018.5	86

Supplemental Table 1. Descriptive values of plasma and microsomal stability of WCDD301 and positive controls in human and mouse.

Supplemental Table 2

Parameter	Results	Reference interval
Blood urea nitrogen (BUN) (mg/dL)	21±2.3	10-79
Creatinine (CREAT) (mg/dL)	0.2±0.1	0.2-0.5
Albumin (ALB) (g/dL)	3±0.1	1.72-3.54
Alanine aminotransferase (ALT) (IU/L)	20.5±9.5	5-394
Aspartate aminotransferase (AST) (IU/L)	99.5±57.5	18-586

Supplemental Table 2. Blood chemistry parameters in non-diabetic NOD mice (mean ± SEM; n=5; 19-week-old) treated with WCDD301.

Supplemental Table 3

Ephrin-A5 Fc (2 nM) WCDD301 (0 µM)	Ephrin-A5 Fc (4 nM) WCDD301 (0 µM)	Ephrin-A5 Fc (6 nM) WCDD301 (0 µM)	Ephrin-A5 Fc (8 nM) WCDD301 (0 µM)
Ephrin-A5 Fc (2 nM) WCDD301 (0.0625 µM)	Ephrin-A5 Fc (4 nM) WCDD301 (0.0625 µM)	Ephrin-A5 Fc (6 nM) WCDD301 (0.0625 µM)	Ephrin-A5 Fc (8 nM) WCDD301 (0.0625 µM)
Ephrin-A5 Fc (2 nM) WCDD301 (0.125 µM)	Ephrin-A5 Fc (4 nM) WCDD301 (0.125 µM)	Ephrin-A5 Fc (6 nM) WCDD301 (0.125 µM)	Ephrin-A5 Fc (8 nM) WCDD301 (0.125 µM)
Ephrin-A5 Fc (2 nM) WCDD301 (0.25 µM)	Ephrin-A5 Fc (4 nM) WCDD301 (0.25 µM)	Ephrin-A5 Fc (6 nM) WCDD301 (0.25 µM)	Ephrin-A5 Fc (8 nM) WCDD301 (0.25 µM)

Supplemental Table 3. Experimental design for assaying the competition between Ephrin-A5 and WCDD301.

Supplemental Table 4

Ephrin-A5 Fc (2 nM) WCDD301 (0 μM)	Ephrin-A5 Fc (2 nM) WCDD301 (0.01 μM)	Ephrin-A5 Fc (2 nm) WCDD301 (0.1 μM)	Ephrin-A5 Fc (2 nM) WCDD301 (1 μM)
Ephrin-A5 Fc (4 nM) - WCDD301 (0 μM)	Ephrin-A5 Fc (4 nM) - WCDD301 (0.01 μM)	Ephrin-A5 Fc (4 nM) - WCDD301 (0.1 μM)	Ephrin-A5 Fc (4 nM) - WCDD301 (1 μM)
Ephrin-A5 Fc (8 nM) WCDD301 (0 μM)	Ephrin-A5 Fc (8 nM) WCDD301 (0.01 μM)	Ephrin-A5 Fc (8 nM) WCDD301 (0.1 μM)	Ephrin-A5 Fc (8 nM) WCDD301 (1 μM)
Ephrin-A5 Fc (16 nM) WCDD301 (0 μM)	Ephrin-A5 Fc (16 nM) WCDD301 (0.01 μM)	Ephrin-A5 Fc (16 nM) WCDD301 (0.1 μM)	Ephrin-A5 Fc (16 nM) WCDD301 (1 μM)
Ephrin-A5 Fc (32 nM) WCDD301 (0 μM)	Ephrin-A5 Fc (32 nM) WCDD301 (0.01 μM)	Ephrin-A5 Fc (32 nM) WCDD301 (0.1 μM)	Ephrin-A5 Fc (32 nM) WCDD301 (1 μM)
Ephrin-A5 Fc (64 nM) WCDD301 (0 μM)	Ephrin-A5 Fc (64 nM) WCDD301 (0.01 μM)	Ephrin-A5 Fc (64 nM) WCDD301 (0.1 μM)	Ephrin-A5 Fc (64 nM) WCDD301 (1 μM)

Supplemental Table 4. Experimental design for assaying the competition between Ephrin-A5 and WCDD301.

Supplemental Table 5

KRBH-11mM (30 min)	KRBH-1mM (60 min)	KRBH-1mM (30 min)	KRBH-11mM (60 min)
Collecting medium-measuring glucagon	Collecting medium-measuring glucagon	Collecting medium-measuring glucagon	Collecting medium-measuring glucagon
Ratio of values (60 min: 30 min)		Ratio of values (60 min: 30 min)	

Supplemental Table 5. Design of glucagon secretion study in the presence or absence of EphA4 inhibitor.

Supplemental Table 6

Donor	Age	Sex	Islet Research Resource ID (RRID)	Years withT1D
1	16	M	SAMN32641505	-
2	34	M	SAMN27361472	-
3	28	M	SAMN31040038	-
4	26	F	UNOSAJC4207	14
5	59	M	UNOSAKDF083	40

Supplemental Table 6. Characteristics of human islet donors provided by the Integrated Islet Distribution Program (IIDP).
